# The influence of care home managers on the implementation of a complex intervention: findings from the process evaluation of a randomised controlled trial of dementia care mapping

**DOI:** 10.1186/s12877-020-01706-5

**Published:** 2020-08-25

**Authors:** R. Kelley, A. W. Griffiths, E. Shoesmith, J. McDermid, E. Couch, O. Robinson, D. Perfect, C. A. Surr

**Affiliations:** 1 Centre for Dementia Research, Leeds Beckett University, Leeds, LS1 3HE UK; 2 Institute of Psychiatry, Psychology and Neuroscience, King’s College London, London, UK; 3 Oxfordshire NHS Trust, Oxfordshire, UK

**Keywords:** Person-centred care, Care homes, Dementia care mapping, Complex interventions, Process evaluation

## Abstract

**Background:**

Many people with dementia live in care homes, where staff can struggle to meet their complex needs. Successful practice improvement interventions in these settings require strong managerial support, but little is known about how managers can support implementation in practice, or what factors support or hinder care home managers in providing this support. Using Dementia Care Mapping™ (DCM) as an example, this study explored how care home managers can support the implementation of complex interventions, and identified factors affecting their ability to provide this support.

**Methods:**

We undertook interviews with 48 staff members (managers and intervention leads) from care homes participating in the intervention arm of the DCM EPIC trial of DCM implementation.

**Results:**

Managerial support played a key role in facilitating the implementation of a complex intervention in care home settings. Managers could provide practical and financial support in many forms. However, managerial support and leadership approaches towards implementation were highly variable in practice, and implementation was easily de-stabilised by management changes or competing managerial priorities. How well managers understood, valued and engaged with the intervention, alongside the leadership style they adopted to support implementation, were key influences on implementation success.

**Conclusions:**

For care home managers to effectively support interventions they must fully understand the proposed intervention and its potential value. This is especially important during times of managerial or practice changes, when managers lack the skills required to effectively support implementation, or when the intervention is complex. It may be unfeasible to successfully implement new interventions during times of managerial or practice instability.

**Trial registration:**

Current Controlled Trials ISRCTN82288852, registered 16/01/2014.

## Introduction

Many people worldwide live in nursing or residential care homes. For example, there are over 2 million people living in long-term care in the US [1] and 450,000 in the UK [2]. Many care home residents have complex needs including dementia, depression, functional dependency, multi-morbidity, mobility or continence issues [1, 3, 4], which can be difficult for care staff to support [5–7]. Practice improvement interventions, often termed ‘psychosocial’ interventions, provide a means through which care homes could improve care quality. However, implementation of psychosocial interventions in health and social care settings is known to be difficult due to their multifaceted nature and multiple components [8]. Intervention delivery involving older adults in long-term care can be particularly challenging and more resource intensive than in other health care settings [9–12], due to residents’ complex needs, low staffing levels, alternative priorities, and insufficient understanding of psychosocial interventions amongst staff [13]. In addition, relatively few studies have explored psychosocial intervention implementation in care homes [14], meaning little is known about how interventions can be supported in these settings [13].

Dementia Care Mapping™ (DCM) [15, 16] is a psychosocial intervention that aims to improve care practices for people living with dementia. It is an observational tool set within a practice development process, to support staff members working in care settings to record and understand experiences of care for people living with dementia, and to use this as a basis for person-centred care planning [17]. It includes a cycle of briefing staff about the DCM process, conducting observations of resident experience using standardised coding frames, analysis of the data, production of a report and feedback to the staff team and action planning for development of care at an individual resident and care home level. This cycle is repeated every 4–6 months to support continual monitoring and development of practice [18]. Despite DCM being used frequently within long-term care settings worldwide [19], recent randomised controlled trials of DCM in care homes have reported heterogeneous results, with implementation issues identified as a likely contributor to the variability in efficacy reported [20]. Developing an in-depth understanding of the barriers and facilitators to successful DCM implementation is therefore crucial, as highlighted by a recent systematic review [20].

To begin exploring implementation issues, three recent DCM trials have included a process evaluation [21–23], although these have been outside of the UK, focused only on nursing homes with qualified nursing staff, and have involved largely atypical, less challenging implementation approaches. For example, researcher-led implementation, cross-over delivery within one provider organisation and use of sites with prior DCM experience or project coordinators [24]. All three process evaluations used small sample sizes and a limited number of implementation sites, with one focusing only on sites that managed to implement DCM [23], limiting transferability to a broader range of care home contexts. From this limited evidence base, challenges to DCM implementation include the time required, staff team resistance to change, and lack of managerial or organisational support [20]. Having an individual with leadership responsibilities for DCM is identified as a key implementation facilitator [23, 24], but beyond this little attention has been paid to understanding how care home managers can support DCM implementation in practice, or to what affects their ability to do so. Broader exploration of general leadership styles which facilitated DCM in three nursing homes [23] identified the value of situational leadership from leaders who were present and knowledgeable, and exploring implementation within one nursing home organisation identified the importance of leaders who were actively involved with and promoted DCM [24]. Neither of these studies explored in-depth the practical features of managerial support that facilitate DCM implementation in routine practice. This state of play accords with wider evidence on successful complex intervention delivery in care home settings; where the importance of managerial support is similarly recognised [13, 25] but insufficiently characterised.

Given the importance of managerial support for effective intervention delivery, greater understanding of how care home managers can support the implementation of complex interventions is required. Using the implementation of DCM as an example, this paper aims to explore what features and actions of managers lend support to complex intervention delivery in care home settings, and what factors affect their ability to offer this support. To do this, we report findings from the process evaluation associated with the DCM EPIC Trial [26, 27] which aimed to evaluate, for the first time, the effectiveness and cost-effectiveness of DCM in UK care homes (nursing and residential) delivered using a pragmatic (‘real world’) approach. During the trial, as with previous trials implementing DCM pragmatically using staff rather than researcher-led implementation, intervention implementation was highly variable with 87% of homes failing to complete the per protocol three cycles of DCM [27] (see methods section for a full description of intervention fidelity). This variability enabled us to explore if, and how, managers influenced DCM implementation across intervention sites.

## Method

### Design

The trial design is reported in full elsewhere [26, 28]. In summary, fifty care homes providing care for people with dementia across three areas of the UK and 942 residents were recruited to the trial. Care homes were randomised on a ratio of 3:2 to intervention or control. Intervention sites were asked to implement DCM alongside usual care and control sites were asked to continue with usual care. In line with standard DCM practice, two staff members (known as mappers) from each intervention site were trained to use DCM and then asked to implement three DCM cycles at 3-months (or as soon as practicable), 8-months and 13-months post-randomisation. Prior to participation, all fifty managers agreed to support DCM implementation if randomised to the intervention, including ensuring mappers were paid but supernumerary on shifts where they were conducting DCM to protect their time, and agreeing time for staff to attend briefing and feedback sessions. In addition to standard DCM practice, the first cycle was supported by a team of external DCM expert mappers. Each expert mapper provided practical support to mappers in several homes, in person and via email/telephone, to support standardised implementation across intervention homes [29]. Further implementation support included the provision of standardised paperwork and reporting templates, sending text message reminders and paperwork ahead of each cycle, and ongoing telephone support from a DCM intervention lead. Despite this additional support, DCM implementation was highly variable across the 31 intervention sites; 23% did not complete any DCM cycles, 52% completed only the first expert-mapper supported cycle, 13% completing two cycles, and only 13% completed the per protocol three cycles within the 16-month study period (see Surr et al. [27] for an overview of intervention fidelity).

The process evaluation followed the Medical Research Council guidelines [30], and aimed to understand implementation fidelity, and barriers and facilitators to DCM implementation, across intervention sites. Care home staff and implementation leads from a sub-group of intervention homes were interviewed about their experiences of DCM implementation. To avoid un-blinding researchers, and to select settings with varying degrees of implementation, interviews took place once all trial outcome data had been collected in each care home.

### Participants

Participants were care home managers, mappers and external expert mappers from 18 of the 31 intervention sites. Purposive sampling was used to select care homes with a range of characteristics that may affect DCM implementation (such as type, location, and size of care home), and across different implementation doses of DCM (0–3 cycles). Where possible in the results, the number of DCM cycles implemented is reported alongside participants’ quotes; for some expert mappers’ quotes this is not possible as they supported and spoke about multiple homes.

In total, 48 participants were interviewed; 17 managers, 25 mappers and 6 expert mappers. Participants were identified by researchers, in conjunction with the manager. Managers and mappers who left the setting during the trial were not interviewed. All participants provided written informed consent. Ethical approval was granted by Bradford Leeds Research Ethics Committee (Ref 13/YH/0016).

### Data collection

Semi-structured interviews were conducted within quiet spaces in each care home by a researcher. Expert mappers were interviewed in their workplace or over the telephone. The length of interviews varied substantially, dependent largely on the interviewee’s level of knowledge about the intervention. Interview length ranged from 5 min (in sites where staff had little or no knowledge of the intervention due to implementation issues) to 1.5 h. Most interviews were conducted individually, with some completed in pairs or small groups (up to 3 participants), based on participant preference.

The interviews were informed by a topic guide (see Additional File 1) designed by the research team in conjunction with the trial lay advisory group. Interviews focused on DCM implementation experiences, discussing each stage of the implementation process, including any barriers and facilitators faced, and the impacts, if any, of DCM. Interviews were audio recorded and transcribed verbatim.

### Data analysis

A Framework Analysis approach [31] was used to identify and develop core themes. The research team developed a coding matrix based on early interview data, which guided and created a structure for further data analysis. The coding matrix focused on experiences of implementing DCM, with, for this paper, a focus specifically on the impacts of managers on implementation.

Using the coding matrix, each transcript was independently coded and analysed by two members of the research team – one from the research hub who had completed trial data collection in the care home and one who had not. The researchers discussed their analysis reached agreement on where quotes should be placed within the framework. The development of coding categories and the framework as a whole continued throughout data analysis, informed by the emerging themes and analytic thoughts of the researchers. Codes and themes were compared and contrasted across care settings and between different types of participants, to develop an in-depth and contextualised understanding of managers’ influences on the implementation of DCM.

## Results

Five themes (summarised in Table 1) focusing on the features and impacts of managers on intervention implementation were identified; *Degree of manager support for the intervention* was crucial, and was influenced by *Managers’ understanding of the intervention, Managers’ choice of intervention leads, Intervention engagement and leadership by managers,* and *Management stability.*

**Table 1:** Summary of main findings

Theme	Summary (supported by quotes in the text)
**Managers’ understanding of the intervention**	To support implementation, Managers’ first needed to understand and see value in the intervention. Despite written and verbal explanations, managers’ understandings were very variable, affecting their ability to support its implementation.
**Degree of manager support for the intervention**	The degree of support for implementation from care home managers, and the value they placed on the intervention, played a crucial role in determining implementation success. Good managerial support included providing time and staffing cover for intervention leads, assisting less confident intervention leads, and supporting engagement with the intervention and resulting practice changes at a practical and financial level, across the care home.
**Managers’ choice of intervention leads**	Managers’ understanding of the skills required to implement a complex intervention, and the availability of staff with the requisite skills, affected managers’ abilities to select appropriately skilled intervention leads. As a result, some intervention leads did not have the required skills or were unprepared for the role and struggled to implement the intervention.
**Management stability**	Managerial stability had a key influence on implementation success, with many homes experiencing one or more management changes during the study. Such changes often signalled difficulties (e.g. in care, staffing or managerial expertise) within the home, and restricted the time new managers had to understand and support the intervention.
**Intervention engagement and leadership by managers**	Engagement with, and leadership of, the intervention varied greatly between managers. Some managers delegated all responsibility for implementation and engaged very little with the process. Others were very engaged, or took ownership by becoming intervention leads although this could be problematic; often possessing the skills, understanding and authority but not necessarily the time to undertake the lead role amid competing priorities.

### Degree of manager support for the intervention

Implementing an intervention focused around practice improvement work with colleagues, within the complex context of care home settings was challenging. The degree of support provided by managers, and the extent to which they valued DCM, played a crucial role in determining how successfully the intervention was implemented:

*“I basically think the manager is so key... in all of those cases [of high or low implementation] it feels like the manager was key” (70001 - Expert Mapper - referring to multiple care homes).*

*“We had a different manager at the time, and she just weren’t interested … wanted us to do it but wouldn’t find us the time to do it.” (50010 - Mapper - 1 cycle completed).*

Managerial support could be provided on multiple levels. Features of good managerial support included protecting time in the staffing rota for mappers to implement DCM and providing staff to cover their usual work, assisting less confident mappers with aspects of implementation such as facilitating feedback sessions or writing reports, helping to engage staff across the home in the intervention and associated practice changes, and supporting (practically and financially) practice changes identified through DCM:

*“I offered to go into it with them, to support them with feedback.” (50019 - Manager - 1 cycle completed).*

*“We worked together … The day that [Mapper’s name] was doing her mapping and her observation, I hired an extra carer, so that extra person would be with the team and let [Mapper’s name] do her work.” (50067 - Manager - 2 cycles completed).*

*“Both managers were really good, if we said we needed time they did slot us into the time [staffing rota].” (50019 - Mapper - 1 cycle completed).*

Whilst some managers were willing and able to offer the types of support identified above, and recognised to need to do so, others did not feel able to provide, or were not perceived as providing, sufficient levels of support for implementation:

*“I’m just disappointed that we weren’t able to continue … because we are only a small home it is quite a large impact on our wage bill to sort of, have people supernumerary.” (10666 - Manager - 0 cycles completed).*

*“I think we had a couple of shifts covered, but … most of the time we had to do it on our days off … Finding the time to do it were our biggest problem.” (50010 - Mapper - 1 cycle completed).*

*“She [the manager] never attended anything. She never came around. She never supported, as far as I could see, the mapper.” (70001 - Expert Mapper - 57065 - 1 cycle).*

As the above quotes demonstrate, some managers felt the time and financial costs associated with supporting their staff to implement DCM could be difficult to maintain. Insufficient managerial support for implementation could lead to unsustainable and demoralising practices such as mappers implementing DCM unpaid in their own time, or partial or failed implementation of important later stages of the intervention, such as report writing, action planning or subsequent DCM cycles.

#### Factors affecting managers’ support

As managers’ support was often crucial to successful implementation, we explored the factors which affected the degree to which managers felt or were able to support DCM implementation. These factors were managers’ understanding of DCM, their engagement with its implementation, their choice of intervention leads, their leadership style and management stability.

### Managers’ understanding of the intervention

If managers were to support implementation of the intervention, it was vital that they understood what the intervention entailed and saw value in it. Although written and verbal explanations of DCM were provided by the research team and the external mappers, managers’ understanding of the intervention was variable. Some managers were able to describe the processes involved and their value, had engaged with its implementation (e.g. by attending briefing sessions or being involved with action planning), and used this knowledge to support implementation:

*“It’s a brilliant tool, and just gives you the time to look and focus on what is going on in your home.” (50067 - Manager - 2 cycles completed).*

*“Having all the [DCM] codes and the level of … how involved they [residents] are with certain things, it just opens your eyes to your residents.” (50069 - Mapper & Manager - 2 cycles completed).*

*“One of the most positive things about mapping is that it gives you a structure to sort of put dementia and dementia care in … it breaks down wellbeing into sensible chunks … it very much gives you the language to actually communicate it to people … Whereas before we would try to improve but we didn’t really know how.” (50018 - Mapper & Manager - 3 cycles completed).*

Many of these managers saw the potential value of DCM for their care setting, or realised the benefits over time, a further driver for supporting its implementation. In contrast, other managers had little understanding of DCM, or awareness of its potential value, despite written and verbal explanations ahead of the trial commencing, having failed to understand or engage with attempts to explain or implement it:

*“I still don’t understand it [DCM] because no one has been able to understand it [explain it] to me fully … Every time I asked them [the mappers] to explain they were struggling. So I never got a full grasp of what it was all about.” (10666 - Manager - 0 cycles completed).*

Whilst managers who understood and valued DCM were willing and able to support its implementation, providing this support was more difficult for managers who did not understand or appreciate the value of DCM:

*“So they would ask me ‘Where does this go?’ and I didn’t go on the [DCM] training so I’m like ‘I’ve no idea where this goes. It’s a massive document [the DCM report], I’m not quite sure.’*” *(50011 - Manager - 1 cycle completed).*

*“I didn’t realise how long things would take and how much effort it would take and that’s probably my fault for not understanding that at the beginning of the process.” (58930 - Manager - 3 cycles completed).*

Misunderstandings around the time required to implement DCM, despite written and verbal information setting this out at recruitment, were one reason for insufficient time being allocated for DCM implementation. In addition, some managers, having observed their staff struggling to implement DCM, concluded that it was too complex and time consuming, reducing their support for ongoing implementation as a result:

*“The reason we pulled out is because they [mappers] couldn’t carry on … with the amount of reports … even with her [expert mapper’s] support. I mean it was like pages and pages and pages.” (50011 - Manager - 1 cycle completed).*

In contrast, despite recognition of the implementation challenges, other managers were enthusiastic about DCM, felt it could drive forwards care improvements in their organisation, and planned to continue its use in their organisation:

“*Going forward I want it to be a regular thing where everybody is mapped every six months.” (50019 - Manager - 1 cycle completed).*

*“We’ve gained more out of it than we’ve put in really, so it has been a positive experience. If not a little traumatic at times!” (58930 - Manager - 3 cycles completed).*

### Managers’ choice of intervention leads

As with many complex interventions, implementing DCM requires a range of skills including IT, written English and effective communication, alongside the confidence and enthusiasm to lead and facilitate changes in workplace practices. The availability of appropriately skilled staff within each care home, and managers’ understanding of DCM, affected their ability to select mappers with the necessary skills. In some homes, managers were able to make considered choices of mappers from willing volunteers, informed by their knowledge of the skills required to implement DCM and the presence of these attributes amongst their staff. In other sites, appropriately skilled mappers were either not selected or were not available:

*“If I look at the whole team there are few other people who would have been possible, academically capable of completing that project [undertaking DCM]. And that’s a difficulty.” (50167 - Manager - 1 cycle completed).*

*“Some managers were really clear [on their choice of mappers] ‘Yep, those two are good communicators, good agents of change, they’ll be good to lead this. For other managers it was completely random.” (70003 - Expert Mapper - referring to multiple care homes).*

It was particularly difficult for managers who knew little about DCM to accurately prepare staff for what implementation would entail or to select staff with the right skills. Mapper selection was sometimes based instead around practicalities such as who was available, likely to continue working in the setting, or would agree to attend the training course:

*“In one case … a new manager … didn’t have a clue about who to nominate … She was just looking at the off-duty and sort of picking names off the off-duty.” (70001 - Expert Mapper - unspecified care home).*

*“From a fairly hard-nosed operational perspective we wanted to pick someone who was likely to be with us at the end of the training, and to continue to benefit from the skills afterwards.” (50018 - Mapper & Manager - 3 cycles completed).*

*“The second nomination [for mapper] that was a bit difficult for me because it was four days away from [geographical area], and that was a bit hard to allocate somebody, because people have got kids, they’ve got other commitments, they do other work.” (50028 - Manager - 1 cycle completed).*

As a result, some mappers were unprepared or un-skilled for the complexity of the role they were required to undertake, and struggled to complete the training course and to implement DCM:

*“They picked two other people to go [on DCM training], and then about three days before they were due to go they backed out. So me and [mapper 2] got slapped into it, not really wanting to do it.” (10666 - Mapper - 0 cycles completed).*

*“There were some people who I think maybe weren’t the right people … They might have been the best of the available … A couple of people … really, really struggled to complete the course … that then potentially creates further problems with being able to write the reports, being able to communicate the information to others.” (70006 - Expert Mapper - referring to multiple care homes).*

The choice of appropriately skilled mappers was particularly limited in homes that were smaller, had high numbers of staff who spoke English as a second language, or lacked qualified nursing staff to become intervention leads.

### Management stability

Managerial stability was a crucial factor in determining implementation success. Over 40% of intervention sites experienced one or more management changes during the 16-months of the trial, which substantially reduced managerial understanding of the intervention:

*“The study was interrupted, the staff that were doing the mapping … left the company, so when I already arrive here [as a new manager], they were not here, and I never had any contact with the mapping.” (50016 - Manager - 0 cycles completed).*

*“I’ve had very little knowledge of it [DCM]. I’ve only been in the home for since November. I haven’t actually seen anything in action.” (50021 - Manager - 2 cycles completed).*

The appointment of new managers, who were sometimes in their first managerial position, typically signalled a time of instability in the setting. For example, new managers could be appointed in response to problems with the quality of care, poor inspection reports (from the Care Quality Commission- CQC), staffing issues, or relationship difficulties between staff and the outgoing management. For new managers, resolving these key issues was their priority, leaving limited time for supporting an intervention they typically knew little about:

*“The manager had left, or the manager was off long-term sick … or there was no manager, or the CQC had been in and they were far too stressed and busy … I heard that a lot.” (70001 - Expert Mapper - referring to multiple care homes).*

New managers could also interrupt DCM implementation by postponing planned intervention dates or by altering practices in the setting, sometimes resulting in staff resignations and further instability. Management changes thus often signalled a time of multiple uncertainties for care homes and presented a significant challenge to successful DCM implementation and sustainability:

*“With the big change that we had with the change in management and everything, that just sent everything all over the place.” (50069 - Mapper & Manager - 2 cycles completed).*

However, on occasions new managers could enhance implementation, for example, if they were more engaged with DCM than the outgoing manager, although, stable management and care quality typically created a stronger foundation for DCM implementation.

### Intervention engagement and leadership by managers

Engagement with, and leadership of, DCM varied greatly between managers. In some sites, managers delegated all responsibility for intervention implementation to their staff and engaged very little with the process:

*“That was my experience, that the managers delegated all aspects to the mappers and didn’t take responsibility for … ensuring the process [of implementing DCM]. I think the odd manager was supportive, again from the office.” (70004 - Expert Mapper - referring to multiple care homes).*

In contrast, other managers took ownership for implementation by becoming intervention leads, as mappers. This approach had several potential advantages. For example, managers could possess the interpersonal and leadership skills required to sensitively lead care improvement discussions and had access to finances to support care improvements that were identified. They also had the authority to protect intervention time in their diaries and to encourage engagement with the intervention and resulting practice improvements throughout the care setting:

*“Some of the briefings [DCM briefings to staff] were really good, and they were generally held by people that had some authority or experience of getting staff together … the best ones were organised by managers, deputy managers … they were great at understanding that everyone should be there... They had the influence to be able to create the spaces to get staff together. They had plenty of un-rostered time … So they could prioritise it.” (70003 - Expert Mapper - referring to multiple care homes).*

Another advantage of managers as intervention leads was their clear understanding of the intervention and the changes required in their care setting, which they were then able to help drive forwards. Managers who clearly understood DCM and the outputs could embed the findings into practice in the care setting, for example, using the findings in training courses for new staff, committing finances to implement practice changes identified through DCM, and highlighting the importance of DCM at an organisational level:

*“[We have] a dementia group which has carers, cleaners, people across the organisation, and you talk to them and you try and actually get them on board. You try and sort of instil in them what person-centred care looks like.” (50018 - Mapper & Manager - 3 cycles completed).*

*“I am more aware [since using DCM] of how staff, certain staff sometimes talk to residents … in the inductions now that we do, we make it really clear about what we want a new member of staff, how we want them to interact … My activities budget is off the scale. But at least I know … there’s stuff going on.” (50069 - Mapper & Manager - 2 cycles completed).*

There were, however, also disadvantages to managers being intervention leads. Although managers benefited from having un-rostered time available, it could be difficult for them to delegate their managerial roles to other members of staff, to attend DCM training or undertake mapping, especially when faced with competing managerial priorities:

*“It was mainly the home, the crisis that the home was in. Every time it sort of came close to me going away … four days out of the building [for DCM training] … it was unfeasible … Knowing the staff we had at the time and the difficulties we had.” (50009 - Manager - 0 cycles completed).*

*“Staff were trying to interrupt “Oh you’ve got to take a phone call about x or y”, or the families were so used to having their attention. Maybe on reflection she might’ve not been the best one to have [as a mapper] … it might have worked a little bit better if it hadn’t been somebody in such a senior role” (50013 - Manager - 1 cycle completed).*

The ability of managers to effectively support DCM implementation was also affected by the different leadership styles they adopted in relation to implementation. Some managers took more active, democratic approaches, sharing ownership of the intervention by involving or supporting their staff in deciding how to give mapping feedback and in feedback delivery, encouraging staff attendance at feedback sessions, and supporting the implementation of practice changes identified through DCM:

*“I think that was partly to do with her [manager in a high implementing home] style of management … She was so able, willing and able to become part of the team when it was required of her, that she was a good role model in every way for them.” (70001 - Expert Mapper - 50069 - 2 cycles completed).*

*“What was amazing in that home [high implementing home] was that the manager was really supportive … the manager would come in and be really enthusiastic. They came to the briefing.” (70005 - Expert Mapper - 10714 - 2 cycles completed).*

In contrast, managers appeared to be less successful intervention leads or supporters when they took an autocratic approach, leading to staff feeling disengaged with the process. Autocratic leadership styles could also affect implementation, with failure to delegate leadership tasks during DCM meaning managers could be repeatedly distracted from mapping and implementation activities:

*“The manager was very frequently distracted and unable to concentrate on the task [mapping] … It felt as if the management structure there was set up in such a way that she was the person that had to deal with everything … If there’s a leak or something gone off the manager gets called away … whereas at [care home name] there was somebody else deployed to do that on mapping days.” (70001 - Expert Mapper - referring to multiple care homes).*

The effects of leadership styles were also evident in the quality of relationships between managers and staff. Whilst many teams and managers worked well together, for others difficulties in relationships could surface during intervention implementation, affecting implementation quality as a result. For example, some managers could be perceived as difficult to approach when staff needed help with implementation. In contrast, other managers had a good rapport with staff and were able to maintain good working relationships throughout the intervention, for example finding effective ways to support potentially difficult discussions with staff around practice improvements:

*“Their [Mappers’] relationship with the manager wasn’t always an easy one and there was lots of ‘Could you talk to her about what we need to be doing?’ … Lots of ‘Oh, she says that to you, but when you’re not here she won’t do it’ … then they’d be ringing me saying ‘We just haven’t been given the time.’ … they didn’t always feel confident to stand up to the manager. (70005 - Expert Mapper - referring to multiple care homes).*

*[Speaking of a manager in a high implementing home] “She’s got very good interpersonal skills … she was never judgmental, she was never critical … if there was a personal detraction [negative staff-resident interaction] … she managed to convey that without making people feel bad about themselves.” (70001 - Expert Mapper - 50069 - 2 cycles completed).*

## Discussion

In summary, managerial support played a central role in facilitating the effective implementation of a complex intervention in care home settings. Managers could provide practical and financial support in many forms. However, in practice, managerial support and leadership approaches were highly variable and implementation was easily de-stabilised by management changes or competing managerial priorities. How well managers understood, valued and engaged with the intervention, alongside the leadership style they employed to support its implementation, were key influences on implementation success.

The importance of good leadership for successful DCM implementation is supported by previous studies of DCM in nursing homes [20]. In taking the first in-depth exploration of the features of effective leadership support for DCM implementation, our findings set out a range of ways in which care home managers can support complex intervention implementation. The features of good managerial support we identified included: financial and practical support for intervention leads and for implementation; ensuring sufficient opportunities and time for implementation and associated practice changes; good relationships between managers and care teams; engagement and shared responsibility for the intervention; and a democratic approach to intervention support and leadership. In developing a framework for the implementation of healthcare interventions, Damschroder et al. [32] highlight twelve influential constructs relating to individual (‘inner’) care settings, of which leadership is one. The emphasis placed on the importance of leadership in our study suggests that implementation research in care home settings may need to consider the particularly influential role that care home managers have on the success or failure of interventions, as well as the interplay between leaders of the care home (i.e. managers) and leaders of the intervention.

Our study is the first to specifically explore factors which enable or prevent care home managers from effectively supporting DCM implementation. Our findings demonstrate that care home managers can play a key role in supporting the on-going challenges of intervention implementation, and of being an intervention lead, providing they have the knowledge and expertise required. This includes strong leadership skills, a good understanding of the intervention, including its value, what supporting it might entail, and who within their organisation might be best placed to support its implementation. We found that when these skills and understanding were lacking, implementation and the selection and support of implementation leads by managers was often challenging.

Management changes, and the resulting instability this could cause, were commonplace and were a particularly significant barrier to intervention implementation, as were changing management priorities (e.g. in response to inspection findings). During our 16-month trial, over 40% of care homes experienced one or more changes in management; annual turnover rates for care home managers in the UK are around 22% [33]. These rates suggest that, whilst manager turnover is commonplace across the UK, the care homes in our study may have experienced higher than average manager turnover and more associated implementation challenges than usual as a result. However, organisational and management changes, such as reorganisations of management or care delivery, are also reported in other DCM trials [8, 24], suggesting such changes should be anticipated and planned for during the implementation of interventions in care homes. Given the importance of managerial support identified by our study, providing additional support for managers during periods of managerial change should be considered by future care home-based intervention studies to reduce implementation issues.

Our study also highlights the importance of ensuring practice improvement interventions focused on care home settings are as easy to understand and implement as possible. Some managers and mappers struggled to manage the workload involved or to understand or convey information about DCM to staff teams, jeopardising their understanding, support and engagement with the intervention. Care home settings are complex in themselves; when implementation is also challenging managers can be disengaged, unenthusiastic about the intervention and unwilling to support it [23, 24]. In contrast, as our findings suggest, when care home staff and managers understand and perceive interventions as likely to improve care for residents they are more motivated to participate in their implementation [34].

Leadership approaches towards the intervention were highly variable in our study, with a democratic approach encompassing higher levels of engagement and sharing of responsibility for implementation appearing to be more effective. The influence of leadership styles, but not their practical features, has been explored in two studies on DCM implementation [23, 24]. They found that successful implementation was dependant on clear leadership support by managers who were situationally present and thus engaged with their staff teams, care practices and the intervention, enabling them to tackle implementation barriers [22]. More widely, other leadership-focused studies in care homes and other care settings indicate that leadership styles and culture influence the implementation of changes in care practices [35–37]. These studies advocate for transformational and consensus-based approaches [37–39], whilst acknowledging that no one leadership style is exclusively advantageous in care settings. Knowing when to use the right style, or combination of approaches, is also important [40, 41]. From our study, and others, it seems clear that supportive and effective leadership is a crucial component for success when implementing interventions in care homes and tackling potential implementation barriers, with our findings setting out practical approaches through which effective leadership may be achieved. Given the complexities and expertise required, and the lack of access to implementation leadership training in care settings [41], future interventions should consider support for managers who may lack the leadership skills required to successfully facilitate intervention implementation themselves.

Finally, our findings highlight how support from, and for, care home managers is required on an on-going basis, throughout intervention implementation. Many intervention leads (mappers) in our study reported a need for ongoing rather than just initial support in relation to intervention implementation, which managers were often well placed to provide. In support of this, van de Ven et al. [8] also reported that mappers found elements of DCM implementation anxiety provoking and required additional support to undertake implementation in practice despite the extensive training provided. Research teams implementing complex interventions therefore need to ensure on-going managerial understanding and support for interventions in care home settings; during their introduction and early implementation, after changes in management, and throughout the implementation period.

### Strengths and limitations

The strengths of this study include the large numbers of interviews undertaken across a range of sizes and types of care home (residential, nursing, and dementia-specialist), the inclusion of a range of views (care home managers and internal and external intervention leads), and a focus on pragmatic rather than atypical delivery of DCM. Triangulation between these different sources, and between sites with differing characteristics and degrees of implementation success, provided a detailed and nuanced exploration of the influence of care home managers on the delivery of a complex intervention. Limitations include high levels of staff and manager turnover during the study, which meant that we were unable to interview some people who had played a key role in implementing the intervention. Undertaking the interviews at the end of the trial is likely to have reduced participants’ recall of earlier parts of the intervention but was necessary to avoid researcher un-blinding, and provided the opportunity to purposefully sample for and explore managerial support in settings with varying degrees of implementation success.

### Practice implications

Careful attention to managerial support for intervention delivery is required when implementing complex interventions in care home settings. High turnover amongst managers means that intervention designs should include strategies for engaging and supporting new managers, alongside attention to ensuring ongoing implementation support from existing managers. Additional support for intervention implementation may be required in sites where managers lack the skills or leadership styles required to effectively support implementation, and especially in settings undergoing significant managerial or practice changes; here the feasibility of implementing any new intervention is questionable and requires careful consideration. It is essential to ensure that managers are engaged with and understand the intervention, including its components and their intended impacts, to help them to identify how they and their staff can best support implementation.

## Supplementary information

**Additional file 1.** Example interview topic guide

## Data Availability

The datasets used and/or analysed during the current study are available from the corresponding author on reasonable request.

## Abbreviations

CQC: Care Quality Commission
DCM: Dementia Care Mapping
