# Supplementary material for: The influence of care home managers on the implementation of a complex intervention: findings from the process evaluation of a randomised controlled trial of dementia care mapping
Source: BMC Geriatr. 2020 Aug 25;20:303. doi: 10.1186/s12877-020-01706-5 (PMC7446218; doi:10.1186/s12877-020-01706-5)
Supplement: Supplementary file 1 — Additional file 1. Example interview topic guide [file 12877_2020_1706_MOESM1_ESM.docx]

**Additional file 1**

**Example interview topic guide:**

**Example Interview Questions**

- How have you found having/supporting Dementia Care Mapping in the care home?
  - Probing about stages of the process (e.g. training, briefing, mapping, feedback, action planning, implementing changes in practice)
- What worked well?
- What were the challenges?
- Experiences of involvement and support from the care home manager
  - Probing around any managerial changes, impacts of managers on implementation
- What impact, if any, has Dementia Care Mapping had?
  - Probing around: impacts on residents, their care, staff, other impacts; whether any impacts have been maintained; if no impacts are reported, why this might be
- Would anything need to change for DCM to work successfully in the care home?

*Footnote: Interview questions varied slightly for each participant group (e.g. manager, mapper, expert mapper); this example provides indicative content.*
